# p21-Activated Kinase 1 Promotes Breast Tumorigenesis *via* Phosphorylation and Activation of the Calcium/Calmodulin-Dependent Protein Kinase II

**DOI:** 10.3389/fcell.2021.759259

**Published:** 2022-01-17

**Authors:** Héctor I. Saldivar-Cerón, Olga Villamar-Cruz, Claire M. Wells, Ibrahim Oguz, Federica Spaggiari, Jonathan Chernoff, Genaro Patiño-López, Sara Huerta-Yepez, Mayra Montecillo-Aguado, Clara M. Rivera-Pazos, Marco A. Loza-Mejía, Alonso Vivar-Sierra, Paola Briseño-Díaz, Alejandro Zentella-Dehesa, Alfonso Leon-Del-Rio, Alejandro López-Saavedra, Laura Padierna-Mota, María de Jesús Ibarra-Sánchez, José Esparza-López, Rosaura Hernández-Rivas, Luis E. Arias-Romero

**Affiliations:** ^1^ UBIMED, Facultad de Estudios Superiores-Iztacala, UNAM, Tlalnepantla, Mexico; ^2^ Departamento de Biomedicina Molecular, Centro de Investigación y de Estudios Avanzados del Instituto Politécnico Nacional (CINVESTAV-IPN), Mexico City, Mexico; ^3^ Division of Cancer Studies, New Hunts House, Guy’s Campus, King’s College London, London, United Kingdom; ^4^ Cancer Biology Program, Fox Chase Cancer Center, Philadelphia, PA, United States; ^5^ Laboratorio de Investigación en Inmunología y Proteómica, Hospital Infantil de México, Mexico City, Mexico; ^6^ Unidad de Investigación en Enfermedades Hemato-Oncológicas, Hospital Infantil de México Federico Gómez, Mexico City, Mexico; ^7^ Facultad de Ciencias Químicas, Universidad La Salle-México, Mexico City, Mexico; ^8^ Programa de Investigación en Cáncer de Mama, Instituto de Investigaciones Biomédicas, UNAM, Mexico City, Mexico; ^9^ Unidad de Bioquímica, Instituto Nacional de Ciencias Médicas y Nutrición Salvador Zubirán (INCMNSZ), Mexico City, Mexico; ^10^ Unidad de Investigación Biomédica en Cáncer, Instituto Nacional de Cancerología-Instituto de Investigaciones Biomédicas, UNAM, Mexico City, Mexico; ^11^ UNe Aplicaciones Biológicas, Laboratorios de Especialidades Inmunologicas, Mexico City, Mexico

**Keywords:** kinase, migration, small molecule inhibitor, synergy, breast cancer

## Abstract

p21-Activated kinase-1 (Pak1) is frequently overexpressed and/or amplified in human breast cancer and is necessary for transformation of mammary epithelial cells. Here, we show that Pak1 interacts with and phosphorylates the Calcium/Calmodulin-dependent Protein Kinase II (CaMKII), and that pharmacological inhibition or depletion of Pak1 leads to diminished activity of CaMKII. We found a strong correlation between Pak1 and CaMKII expression in human breast cancer samples, and combined inhibition of Pak1 and CaMKII with small-molecule inhibitors was synergistic and induced apoptosis more potently in Her2 positive and triple negative breast cancer (TNBC) cells. Co-adminstration of Pak and CaMKII small-molecule inhibitors resulted in a dramatic reduction of proliferation and an increase in apoptosis in a 3D cell culture setting, as well as an impairment in migration and invasion of TNBC cells. Finally, mice bearing xenografts of TNBC cells showed a significant delay in tumor growth when treated with small-molecule inhibitors of Pak and CaMKII. These data delineate a signaling pathway from Pak1 to CaMKII that is required for efficient proliferation, migration and invasion of mammary epithelial cells, and suggest new therapeutic strategies in breast cancer.

## Introduction

Breast cancer is one of the most common malignancies among women and is the fifth leading cause of cancer-related deaths (Siegel et al., 2012; Sung et al., 2021). The median age at diagnosis is 62 years, and approximately 20% of the patients are diagnosed before the age of 50. In general, 60% of breast cancers are found as a localized tumor that is treated with lumpectomy or mastectomy (Siegel et al., 2012). Breast cancer is a complex and heterogeneous disease that can be divided into four intrinsic subtypes: Luminal A (50–60% of breast tumors), Luminal B (15–20% of breast tumors), Her2+ (15–20% of breast tumors) and triple negative breast cancer (TNBC, 15–20% of breast tumors), which are mainly defined by the expression of estrogen receptor (ER), progesterone receptor (PR) and Her2, as well as in Ki-67, EGFR and basal cytokeratines status (Dai X. et al., 2015).

Signal transduction is a fundamental process in the development and progression of cancer through their role in regulating pro-survival and anti-apoptotic signaling pathways. Some of the most prominent signaling networks affected in breast cancer include, the phosphoinositide 3-kinase (PI3K)/Akt/mTOR, Ras/RAF/MEK/ERK, and Src/Fak pathways (Marcotte and Muller, 2008).

p21 activated kinases (Paks) are downstream effectors for the small GTPases Cdc42 and Rac that regulate the activity of the PI3K/Akt, Ras/RAF/MEK/ERK, and Src/Fak networks (Arias-Romero and Chernoff, 2008; Radu et al., 2014). In addition, it has been shown that one member of the Pak family, Pak1, is amplified and/or overexpressed in different types of cancer, including 25–30% of breast tumor samples and cancer cell lines (Radu et al., 2014). For instance, it has been reported that Pak1 is necessary for the activation of Akt, either acting as a scaffolding protein that links Pdk1 to Akt or directly phosphorylating Akt at the residue Ser473 (Higuchi et al., 2008; Mao et al., 2008). In the Ras/RAF/MEK/ERK pathway, Pak1 phosphorylates both, c-Raf at S338 and MEK1 at S298, sites that are essential for full activation of MAPK signaling (Beeser et al., 2005). In addition, Pak1 also acts downstream in the Src/Fak pathway, as an effector for the small GTPase Rac1 (Zhao et al., 2000). However, the role of Pak1 in tumorigenesis, and the particular signaling pathways affected, are not completely understood.

In this work, we show that Pak1-deficient breast cancer cells showed a dramatic reduction in CaMKII phosphorylation at residue T287, which is important for the activity of this kinase. In addition, using *in silico* and *in vitro* kinase assays, we showed that Pak1 interacts with and phosphorylates CAMKII not only at T287, but also at T277. Moreover, we demonstrated that both, pharmacological inhibition of Pak1 activity or reduction of Pak1 expression through siRNA-mediated assays, significantly reduced the phosphorylation of CaMKII at T287. Conversely, the expression of a rapamycin-activatable Pak1 increased its phosphorylation levels. In addition, Pak1 and CaMKII are co-expressed in breast cancer cell lines and using a human breast cancer tissue microarray (TMA), we observed a significant correlation between the expression levels of these two kinases. Next, we showed that in a 3D cell culture setting, the combination of anti-Pak and anti-CaMKII agents has a synergistic inhibitory effect on cell proliferation, and induced apoptosis more potently in Her2 positive and TNBC cells, as well as an impairment in cell migration and invasion. Finally, we demonstrated that the combination of small molecule inhibitors targeting Pak1 and CaMKII significantly delayed the tumorigenesis of TNBC cells in a xenograft setting. These data delineate a signaling pathway from Pak1 to CaMKII that is required for efficient proliferation, migration and invasion of mammary epithelial cells, and suggest new therapeutic avenues for the treatment of TNBC.

## Materials and Methods

### Antibodies and Reagents

Antibodies used for western blot included anti Pak1, phospho-Pak1 (Ser199/204), CaMKII and phospho-CaMKII Thr287 pan-antibodies, myc-tag, cleaved caspase-3, PCNA and GAPDH from Cell Signaling Technology (Boston, MA. United States). Anti-GFP was from Santa Cruz Biotechnology (Dallas, TX. United States), and Anti-phospho-Threonine Antibody, clone 20H6.1, was from Sigma-Aldrich (St. Louis, MO. United States).

### DNA Constructs and siRNA

The pCMV6M-Pak1 plasmid (Sells et al., 1997), was a gift from Jonathan Chernoff (Addgene plasmid 12209). The uniRapR-Pak1 mammalian expression vector (Dagliyan et al., 2017), was a gift from Klaus Hahn. The CaMKII*γ* cDNA was amplified by PCR using the pDONR223-CAMK2G vector (Addgene plasmid 23409) as a template and cloned into the HindIII/EcoRI restriction sites of pEGFP-C1 (Clonetech. Mountain View, CA. United States). The sequence encoding the regulatory domain (RD) of CaMKII*γ* (aminoacids 212–317) was amplified by PCR and cloned into the BamHI/EcoRI sites of pGEX-6P-2 (GE Healthcare. Braunschweig, Germany). The pGEX-6P-2-CaMKII*γ*RD T277A, T287A and the double mutant T277/287A were generated by site-directed mutagenesis with the Quick Change kit (Stratagene. La Jolla, CA. United States). The SMARTpool siRNAs targeting human Pak1 were obtained from Dharmacon (Lafayette, FL. United States).

### Cell Culture

Primary breast cancer cells derived from MMTV-*Neu:Pak1* ^+/+^ and MMTV-*Neu:Pak1* ^−/−^ animals were manteined in low calcium medium supplemented with 5% horse serum (Arias-Romero et al., 2013). MCF10A, MCF-7, SK-BR3, and MDA-MB-231 cells were obtained from American Type Culture Collection (Manassas, VA, United States), MBCDF-B4 cells were a kind gift from Dr. María de Jesús Ibarra-Sánchez (Esparza-López et al., 2016). MCF10A cells were maintained in DMEM/F12 (Gibco BRL) supplemented with 10% FBS, 20 ng/ml EGF (Harlan Bioproducts. Indianapolis, IN. United States), 10 μg/ml insulin (Sigma-Aldrich. St. Louis, MO. United States), 1 ng/ml cholera toxin (Sigma-Aldrich. St. Louis, MO. United States), 100 μg/ml hydrocortisone (Sigma-Aldrich. St. Louis, MO. United States), 50 U/mL penicillin, and 50 μg/ml streptomycin. MCF-7, MDA-MB-231and MBCDF-B4 cells were grown in DMEM supplemented with 10% FBS, 50 U/mL penicillin, and 50 μg/ml streptomycin. SK-BR3 were grown in McCoy’s 5A supplemented with 10% FBS, 50 U/mL penicillin, and 50 μg/ml streptomycin.

For 3D cell cultures, 1500 MCF7, SK-BR3 or MDA-MB-231 cells were plated atop reconstituted basement membrane in eight-well slide chambers as described (Debnath et al., 2003). Cells were treated on day 5 with vehicle (control), FRAX-1036, 1 µM KN93 of FRAX-1036 and KN93 respectively, fresh cell culture media supplemented with the corresponding inhibitors every other day, and 3D cultures were fixed on day 15. The 3D cell cultures were then stained with Oregon Green Phalloidin (Thermo Fisher Scientific); 4′, 6 diamidino-2-phenylindole (DAPI) (Thermo Fisher Scientific) and anti–PCNA, or anti-cleaved PARP-1 (Asp214) antibodies (Cell Signaling Technology). Samples were analyzed under fluorescence microscopy using a Zeiss LSM710 Duo confocal microscope. Percentage of PCNA–positive, and anti-cleaved PARP-1–positive cells were scored on the basis of assessment of 30 spheroids per well. Bar, 50 mm. Results were represented as mean ± S.E. Significance (*p*-value) between cell lines was determined using the Student *t*-test. *p < 0.05; **p < 0.01.

### Phospho-specific Protein Microarray Analysis

The cancer signaling phospho-antibody arrays were purchased from Full Moon Biosystems, Inc. (Sunnyvale, CA. United States). Protein microarray analysis was performed following the manufacturer’s suggested protocol. Briefly, 100 μg of cell lysates were diluted in 50 μL of reaction mixture and labeled with biotin in 10 μg/μL N,N-dimethyformamide. The biotin-labeled proteins were diluted 1:20 in coupling solution before applying to the array for conjugation. To prepare the antibody microarray, it was blocked with blocking solution for 30 min at room temperature, rinsed with Milli-Q grade water, and dried with compressed nitrogen. Next, the array was incubated with the biotin-labeled proteins at 4°C overnight, the arrays were washed three times with 60 ml of wash solution, and the conjugated-labeled proteins were detected using Cy3-streptavidin. Scanning, quantification, and data normalization were done using a G4900DA SureScan Microarray Scanner System and Feature Extraction v12.0 (Agilent Technologies). The extracted data were normalized and analyzed with the Subió platform (Subió Inc).

### Model of the Binding Complex of the Pak1 and Pak2 Kinase Domain and CaMKII*γ* Peptide I and II

To generate a model that could show the interaction of CaMKII*γ* and its derived peptides I (sequence: LKHPWVCQRSTVASMMHRQET where the underline Thr11, corresponding to Thr 277, which was identified as a phosphorylation target) and II (sequence: STVASMMHRQETVSLRKFNAR, where the underlined Thr12, which corresponds to Thr287 in CaMKII*γ*, which was also identified as a phosphorylated residue), we decided to carry out peptide-protein and protein-protein docking experiments followed by molecular dynamics simulations. First, PEPstrMOD online platform (Singh et al., 2015) was used to predict the structure of the sequences of both peptides while CaMKII*γ* structure was retrieved from the Protein Data Bank (PDB) (Berman et al., 2000) with the accession code PDB ID: 2V7O (Rellos et al., 2010). The activated Pak1 kinase domain in complex with ATP structure was also downloaded from the PDB with the accession code PDB ID:3Q53 (Wang et al., 2011). For Pak2, currently there is no available tridimensional structure in the PDB, in consequence the model was generated using PAK1 as template with the homology modelling module implemented as part of Yasara Structure v.18.4.24. For the docking experiments, ClusPro online server https://cluspro.bu.edu/(Kozakov et al., 2013; Kozakov et al., 2017; Vajda et al., 2017; Desta et al., 2020) was used. ClusPro 2.0 generates conformations based on different desolvation and electrostatic potential; those conformations are categorized through clustering.

Molecular dynamics simulations were carried out in duplicate using Yasara Structure v. 18.4.24 (Krieger and Vriend, 2014; Krieger and Vriend, 2015) using the AMBER 14 force field using a previously reported protocol (Vivar-Sierra et al., 2021). Briefly, each complex was embedded within a TIP3 water box with 10 Å to the box boundary. Periodic boundary conditions were considered. The temperature was set to 298 K, pH to 7.4, with the addition of sodium and chlorine ions for charge neutralization. A Particle Mesh Ewald (PME) algorithm with a cut-off radius of 8 Å was applied. Steepest descent energy minimization was performed, and then a total simulation time of 100 ns with a time step of 2.5 fs was carried out, recording snapshots at intervals of 500 ps. The analysis of the resulting trajectories was performed with a script included as part of Yasara software and included the root mean square deviation (RMSD) and the distance of the phosphorus atom in the *γ*-phosphate group of ATP to the oxygen atoms of the side-chain threonine residues (O_(Thr)_- *γ*P) identified as the Pak1 or Pak2 phosphorylation targets.

### Tissue Microarray (TMA)

190 breast cancer specimens of at least 100 mg were obtained from the Fox Chase Cancer Center tumor core at the time of surgery from each patient per an Institutional Review Board approved protocol. The tissues were validated as invasive mammary carcinomas by a pathologist, immediately frozen in liquid nitrogen and stored at −80°C. The archived H&E slides used for diagnosis were verified by the pathologist for confirmation of diagnosis and selection of appropriate paraffin-embedded tissue blocks for the construction of TMAs. Slides with the tissue of interest were selected and mapped for construction of the TMA blocks using a 1.5 mm punch size.

### Immunohistochemistry

The IHC of all the biomarkers analyzed in this study were performed as previously described (Zapata-Tarres et al., 2019). Briefly, serial sections of the breast TMA were deparaffinized with xylene, and then rehydrated with graded ethanol. Antigen retrieval was performed using sodium citrate (0.01 M, pH 6.0). Endogenous peroxidase activity was inhibited with methanol and 3% hydrogen peroxide. Non-specific binding was blocked by immersing the sections in universal blocking solution and 1% bovine serum albumin for 60 min, the tissue slices were incubated overnight at room temperature with an anti-CaMKII antibody (dilution 1:1,000), Pak1 antibody (dilution 1:750). The slides were washed and incubated with specific secondary antibodies conjugated to horseradish peroxidase (HPR) (Vector. Burlingame, CA. United States) or the secondary antibody anti-goat conjugated to HRP (GBI Labs. Bothell, WA. United States) for hENT1 and the signal was generated by the addition of diaminobenzidine (Vector. Burlingame, CA. United States). The reaction was stopped, and samples were counterstained with hematoxylin. The tissues were dehydrated, and the preparations were covered with resin and dried at room temperature. In order to diminish the inter-assay variability, immunohistochemistry analysis was performed at the same time in a single experiment for each biomarker.

For the tumor mice tissue, we follow the same method describe below using anti-PCNA dilution 1:1,000 from Genetex (Irvine, CA. United States), anti-cleaved Caspase 3 antibodies dilution 1:500 from Millipore (Burlington, Massachusetts. United States), anti phospho-Pak1 Thr212 and anti phospho-CaMKII Thr287, dilution 1:200 both from ThermoFisher (Waltham, Massachusetts. United States). Protein expression analysis of each tissue section was performed by digital pathology using a ScanScope CS digital processor (Aperio, San Diego, CA. United States).

### Digital Imaging Analysis

Immunohistochemically stained samples were analyzed as previously described (Zapata-Tarres et al., 2019). Briefly, immunostained sections for each protein were digitized at a 40x magnification using an Aperio ScanScope CS (Leica BioSystems. Nussloch, Germany). The Aperio ScanScope CS obtains 40x images with a spatial resolution of 0.45 μm/pixels. The images were reviewed using the ImageScope software (Leica BioSystems. Nussloch, Germany). Once the areas were annotated, they were sent for automated image analysis using Spectrum Software (Leica BioSystems. Nussloch, Germany). For tissue intensity, an algorithm was developed to quantify the total CamKII and Pak1 expression. The output from the algorithm returns a number of quantitative measurements, namely, the intensity, concentration and percentage of positive staining. Quantitative scales of intensity and percentage were categorized into 4 and 5 classes, respectively, after the cut-off values were determined. The intensity of staining was categorized as 0 (no staining), 2+ (moderate) or 3+ (strong). The final IHC score was calculated from a combination of the intensity of total or nuclear expression and percentage scores.

### Immunofluorescence and Image Analysis

Cells were seeded in coverslips, fixed in 4% paraformaldehyde and permeabilized with 0.2% Trton X-100, blocked and incubated with primary antibodies for 2 h at room temperature. Cells were then washed three times with PBS and incubated with Alexa Fluor 488 or 594-conjugated secondary antibodies (Thermo Fisher Scientific. Waltham, MA. United States). Stained cells were imaged using a Leica TCS SP8 confocal microscope. Co-localization was quantified using ImageJ and Coloc2 plugin software (NIH).

### Immunoblotting and Co-Immunoprecipitation

Breast cancer cells were lysed in RIPA buffer (20 nM Tris-HCL pH 7.4, 150 nM NaCl, 1 mM EDTA, 1% Triton X-100, 0.5% SDS, 1% sodium deoxycholate, 1X protease inhibitor Cocktail and 1X PhosSTOP (Sigma-Aldrich. St. Louis, MO. United States). Immunoblots (on Immobilon-P membranes (Millipore. Burlington, Massachusetts. United States) were blocked in 5% nonfat dried milk in TBS-Tween-20 0.5% or 1% BSA, incubated primary and secondary antibodies, and visualized using enhanced chemiluminescence reagents (ECL, Amersham Pharmacia. Buckinghamshire. United Kingdom). All the antibodies were used at concentrations as recommended by the supplier.

For co-immuniprecipitation cells were lysed for 30 min in PBSCM buffer (100 mM Na_2_PO_4_, 150 mM NaCl, pH 7.2, 1 mM CaCl_2_, 1 mM MgCl_2_ and 5 µM ATP), homogenized and centrifuged at 13,500rpm for 10 min at 4°C. The supernatants were recovered, clarified and incubated with primary antibodies or mouse or rabbit IgG isotype control antibodies for 4 h at 4°C, and incubated with Protein G Sepharose beads (GE Healthcare. Braunschweig, Germany). The immune complexes were washed three times with PBSCM buffer and the bound material was eluted using sample buffer for 5 min at 90°C. The eluate was resolved on 10% SDS–PAGE and analyzed by immunoblot.

### Wound Healing Assay

Cells were seeded in 6-well tissue culture plates to form a monolayer and treated with 10 μg/ml of mitomycin c and the relevant small-molecule inhibitors. Wounds were created with a 200 µL pipette tip, and wound closure was observed at different time points within the scrape line (0 h, 24 h), and the extent of wound closure was determined with a Leica DM IL LED inverted microscope (Wetzlar, Germany). The data was analyzed with the ImageJ and GraphPad Prism 8.0 software.

### Transwell Migration Assay

The *in vitro* cell migration assay was performed using 8 µm-pore size Transwell chambers (BD Bioscience. Franklin Lakes, NJ. United States) according to the manufacturer’s instructions. Briefly, 2.5 × 10^4^ cells were treated with Pak and/or CaMKII inhibitors and added to the top chamber in serum-free medium. The bottom chamber contained cell culture medium supplemented with the corresponding small-molecule inhibitors and 10% FBS. Cells were incubated for 24 h at 37°C, fixed with 70% methanol for 15 min and stained with crystal violet (0.2%) for 15 min, and counted in three separate, random view fields under a Leica DM IL LED inverted microscope (Wetzlar, Germany).

### Random Migration Assay

5 × 10^4^ MDA-MB-231 or MBCDF-B4 cells were grown in DMEM 10% FBS, treated with 20 mM Hepes and placed on a heated (37°C) stage of an Olympus IX71 microscope. Images were collected using a Retiga SRV CCD camera every 5 min for 16 h using Image-Pro Plus software. All the acquired time-lapse sequences were displayed as movies and cells were tracked using ImageJ. Mathematical analysis was performed using Chemotaxis and Migration Tool 2.0 where the mean cell migration speed and accumulated distance is calculated for each cell and this data is used to calculate a mean cell migration speed for each group of cells. Statistical significance was calculated using ANOVA.

### Invadopodia Assay

The invadopodia formation assays was performed using the QCM™ Gelatin Invadopodia Assay (Millipore. Burlington, Massachusetts. United States) according to the manufacturer’s instructions. Briefly, coverslips were coated with poly-L-lysine for 20 min, washed three times with PBS and incubated with glutaraldehyde:PBS for 15 min. The coverslips were placed on mix of fluorescently labeled gelatin. Cells were treated with vehicle, FRAX-1036 (MedChem Express, Monmouth Junction, NJ. United States) and/or KN93 (Santa Cruz Biotechnology. Dallas, TX. United States), seeded onto the gelatin-coated coverslips and incubated for 24 h at 37°C. Then, the cells were fixed and permeabilized, and the cytoskeleton and nuclei were stained with Oregon Green-Phalloidin and DAPI respectively. The samples were observed in a Leica TCS SP8 confocal microscope (Wetzlar, Germany). and the results were analyzed using LAS X ® software.

### Tumor Xenografts

The Institutional Animal Care and Use Committee (IACUC) of CINVESTAV approved all animal experiments (protocol number: 0,307-2). Four to 6-week-old inbred BALB/c-nu/nu female mice were injected with MDA-MB-231 cells (5 × 10^6^ in 0.3 ml of rBM) into the mammary fat pad of each mouse. FRAX-1036 was formulated in 20% of 2-Hydroxypropyl-*β*-cyclodextrin in 50 mM citrate buffer (pH 3.0) and administrated to mice receiving a dose of 30 mg/kg/day *via* oral gavage. KN93 was reconstituted in corn oil, and mice were intraperitonially injected with a dose of 50 mg/kg/day, in the combination groups, the compounds were given with 4–6 h interval. At completion of all xenograft studies mice were culled, the tumors were collected and their volumes estimated with the following formula: volume = (a2 X b)/2, where a = short and b = long tumor lengths, respectively, in millimeters.

### Statistics

Relationships between the expression levels of different markers evaluated in the TMA were examined using Spearman rank correlation. Statistical analysis was conducted using a two-way ANOVA or the unpaired Student *t* test except for survival. Values of *p* < 0.05 were considered significant. For the xenograft studies, treatment cohorts were analyzed by one-way analysis of variance using the Prism software package (GraphPad Software. San Diego, CA. United States).

## Results

### CaMKII Is a Novel Substrate of Pak1

**FIGURE 1:**
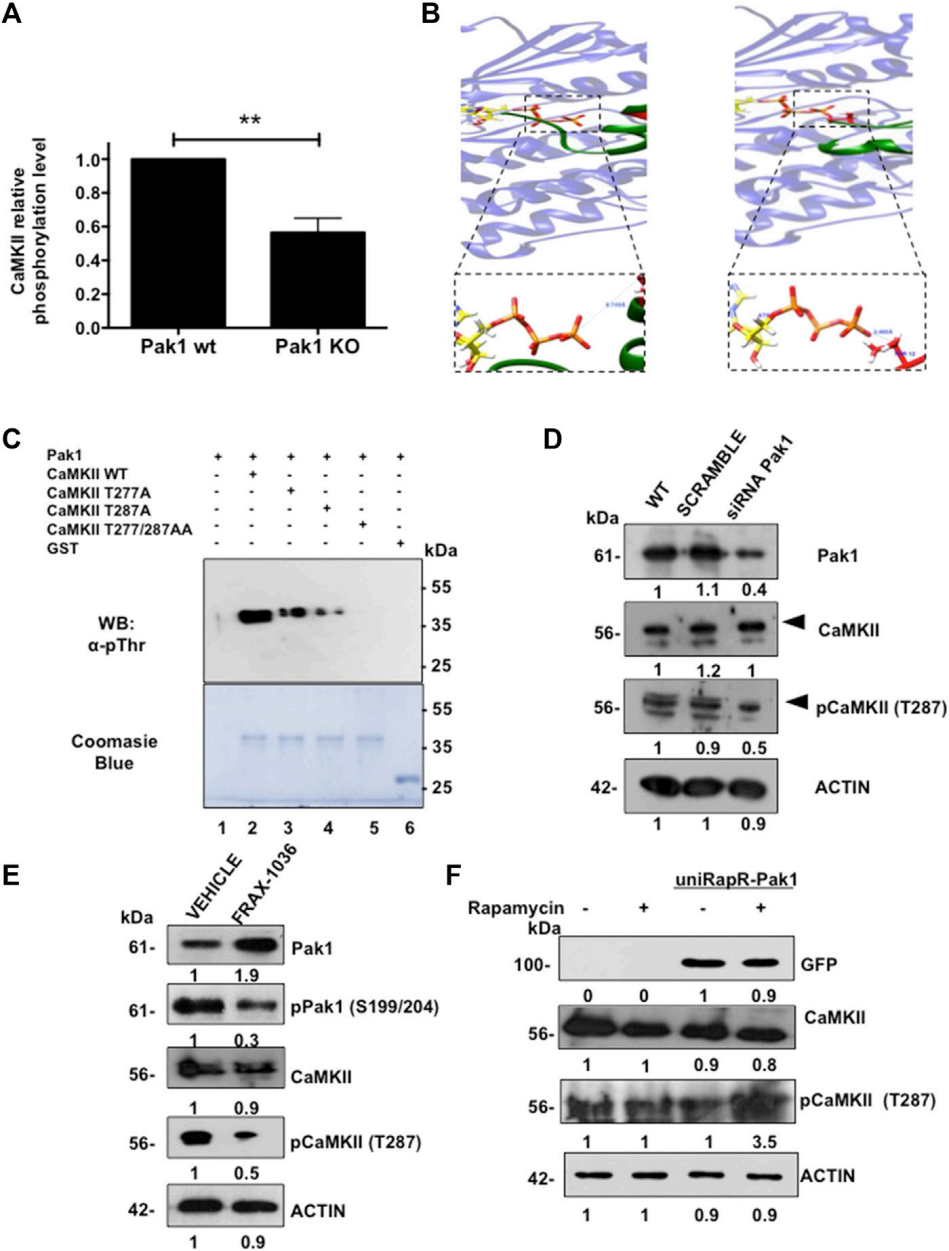
Identification of CaMKII as a Pak1 novel substrate. **(A)** CaMKII phosphorylation is reduced in Pak1-deficient cells. Results of the phospho-antibody array quantification for CaMKII are presented as changes in phosphorylation between control and Pak1-deficient cells. **(B)** Visualization of the complex of peptide I **(left)**, and peptide II **(right)** with ATP-bound Pak1. The boxed panels show a closer view of the O_(Thr)_-*γ*P interaction for peptides I and II, respectively. **(C)** *In vitro* kinase assay with wild type, T277A, T287A or the double mutant T277/287A recombinant GST-tagged CaMKII*γ* RD as substrates of Pak1. The substrate phosphorylation was detected by western blot (upper panel), whereas the total amount of substrate in each reaction is shown by Coomassie blue staining (bottom panel). **(D)** Pak1 knock down with siRNAs negatively affects the activation of CaMKII in breast cancer cells. Numbers indicate fold expression or phosphorylation change relative to control cells. **(E)** Pak1 pharmacological inhibition negatively affects the activation of CaMKII in breast cancer cells. Numbers indicate fold expression or phosphorylation change relative to control cells. **(F)** The expression of a rapamycin-activatable Pak1 promotes the phosphorylation of CaMKII in breast cancer cells. MDA-MB-231 cells were mock transfected or transfected with uniRapR-Pak1 expression vector and incubated with vehicle or rapamycin. Ectopic Pak1 expression was detected with anti-GFP antibodies. Numbers indicate fold expression or phosphorylation change in CaMKII relative to control cells.

Pak1 occupies a central position in oncogenic signaling. In the last decade, several substrates of this kinase have been validated (Radu et al., 2014). However, additional substrates that mediate the oncogenic effects of Pak1 in different types of cancer remain to be identified. In order to examine signaling proteins that might be regulated by Pak1 we used a phospho-antibody array, which contains several dozen of breast cancer-relevant phospho-protein specific antibodies. This array was probed with lysates from wild type and Pak1 deficient breast cancer cells derived from murine tumors (Arias-Romero et al., 2013). The results showed that phosphorylation of several Pak1 direct and indirect substrates, including ERK, c-Raf, Akt and *β*-catenin were significantly reduced in Pak1 deficient cells (Supplementary Figure S1A). Interestingly, one of the proteins showing a significant reduction in phosphorylation levels was the Calcium/Calmodulin-dependent kinase, CaMKII (Figure 1A), a protein that has recently been associated to breast cancer progression (Chi et al., 2016; Wang et al., 2017). Since CaMKII family is a highly conserved group of proteins composed of four members; *α*, *β*, *γ* and *δ*, we analyzed the relative expression at mRNA level of the four CaMKII isoforms using TCGA data. We observed that only CaMKII*γ* expression is statistically higher in tumor samples than in adjacent normal tissue (Supplementary Figure S1B). In addition, the expression of Pak1 and CaMKII in the non-transformed mammary epithelial cell line MCF10A, and in the breast cancer cell lines MCF7 (luminal), SK-BR3 (Her2 positive) and MDA-MB-231 (triple negative) was assessed by western blot. Interestingly, the cell lines with higher levels of CaMKII correspond to the Her2 positive and TNBC subtypes (Supplementary Figure S1C).

Then, we performed a Group-based phosphorylation site (GPS) analysis (Zhou et al., 2004) of CaMKII*γ* primary sequence. This analysis revealed the presence of two putative Pak1 phosphorylation sites located at T277 and T287. In order to determine if Pak1 phosphorylates CaMKII*γ* in these two residues, we performed peptide-protein docking experiments followed by molecular dynamics simulations. First, PEPstrMOD online platform (Singh et al., 2015) was used to predict the structure of the sequences of the CaMKII*γ* peptide I (LKHPWVCQRSTVASMMHRQET) and peptide II (STVASMMHRQETVSLRKFNAR), where the underlined T residues correspond to T277 and T287 respectively. Next, we generated the interaction models by docking in the ClusPro online server (Kozakov et al., 2017; Alekseenko et al., 2020), using the Pak1 kinase domain in complex with ATP (PDB ID:3Q53) (Wang et al., 2011). The molecular dynamic simulations results indicated that the distance from the oxygen atoms of the T277 and T287 residues to the phosphorous atom of the *γ*-phosphate of ATP (O_(Thr)_-*γ*P) is 6.75 Å and 3.5 Å respectively (Figure 1B; Supplementary Figure S1D), suggesting that Pak1 can phosphorylate CaMKII on the aforementioned residues. In order to assess if the closely related and broadly expressed Pak2 kinase also phosphorylates CaMKII, the structure of this Pak family member was generated by homology modeling using Pak1 structure as template. For the Pak2 models, the hydroxyl groups of the potential phosphorylation sites were located at a greater distance than in the case of Pak1, 17.5 Å for peptide I and 7.5 Å for peptide II, suggesting that CaMKII is a better substrate for Pak1 than for Pak2 (Supplementary Figure S1D). In addition, we explored if CaMKII is a Pak1 substrate by using an *in vitro* kinase assay. The wild type, T277A, T287A or the double mutant T277/287A GST-tagged regulatory domain of CaMKII*γ* (212-317 aa) was incubated with purified Pak1. We observed a dramatic reduction in the phosphorylation levels of the T277 (50%) and T287 (70%) mutants when compared to the wild type CaMKII*γ* fragment. In contrast, the double mutant T277/287 was not phosphorylated by Pak1, suggesting that CaMKII is a substrate of Pak1 (Figure 1C).

Finally, we validated the Pak1 phosphorylation of CaMKII in a physiologically relevant setting. Since MDA-MB-231 cells showed a high expression of both kinases, we transfected these cells with siRNAs targeting Pak1. Consistent with *in vitro* studies, we noticed a substantial reduction in the phosphorylation of CaMKII T287. Similarly, the pharmacological inhibition of Pak1 with the small-molecule inhibitor FRAX-1036 resulted in a reduction in CaMKII phosphorylation (Figures 1D,E). Moreover, the knockdown of Pak2 with siRNAs had no effect on the phosphorylation levels of CaMKII, suggesting that Pak1, but not the closely related Pak2 phosphorylates CaMKII (Supplementary Figure S1F). In contrast, we observed a significant increase in the phosphorylation levels of CaMKII (Figure 1F), when we transfected MDA-MB-231 cells with a rapamycin-activatable Pak1 (Dagliyan et al., 2017). These findings strongly indicate that the T287 residue of CaMKII is phosphorylated by Pak1.

### Pak1 Interacts With CaMKII

**FIGURE 2:**
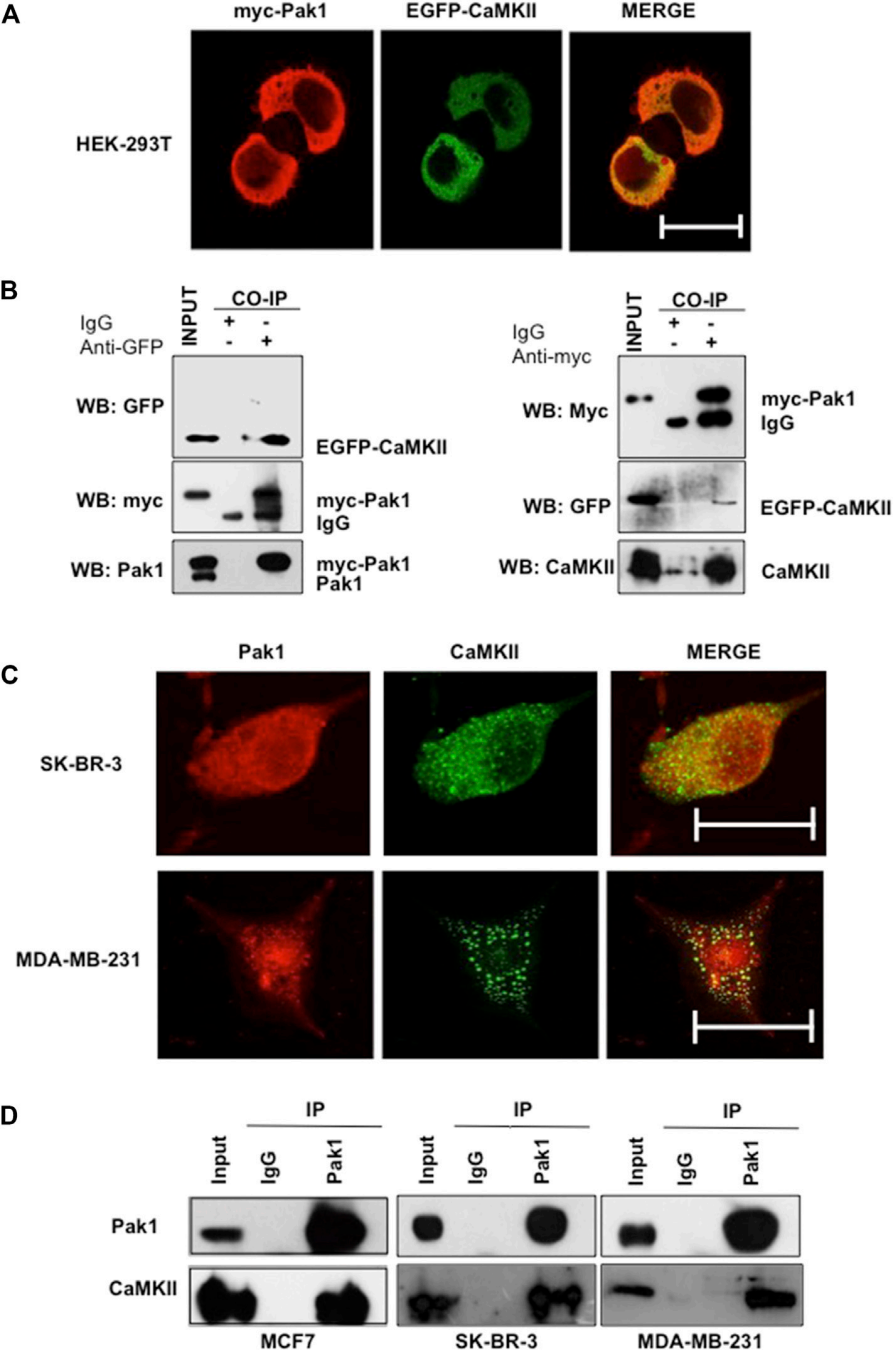
Pak1 interacts with CaMKII in a cellular context. **(A)** HEK293T cells were co-transfected with expression vectors encoding GFP-tagged CaMKII and myc-tagged Pak1. Cells were analyzed by confocal microscopy acquiring a Z-stack of confocal optical sections at 0.2 μm steps. 3D confocal images were post-treated by deconvolution. A 0.4 μm-thick medial stack is shown. Bar = 10 μm. **(B)** Co-immunoprecipitation of Pak1 and CaMKII. HEK293T cells were co-transfected with pCMV6M-Pak1 and pEGFPN1-CaMKII vectors to express myc-tagged Pak1 and GFP-tagged CaMKII respectively. Cell lysates were subjected to immunoprecipitation with anti-myc, anti-GFP or isotype control IgG antibodies. The presence of myc-Pak1 and GFP-CaMKII in cell extracts prior to immunoprecipitation was assessed using anti-myc and anti-GFP antibodies (Input). **(C)** SK-BR3 and MDA-MB-231 cells were transfected with GFP-tagged CaMKII, endogenous Pak1 was stained with anti-Pak1 antibodies. Bar = 10 μm. **(D)** Co-immunoprecipitation of endogenous Pak1 and CaMKII. MCF7, SK-BR3 and MDA-MB-231 cell lysates were subjected to immunoprecipitation with anti-Pak1, anti-CaMKII or isotype control IgG antibodies. The presence of Pak1 and CaMKII in cell extracts prior to immunoprecipitation was assessed using specific antibodies (Input).

Given that Pak1 phosphorylates CaMKII *in vitro*, we wonder whether both proteins interact in a cellular context. To this end, HEK293T cells were co-transfected with myc-tagged Pak1 and GFP-CaMKII expression vectors and their co-localization was analyzed by confocal microscopy. The results showed that Pak1 and GFP-CaMKII proteins co-localize in the cytoplasm of HEK293T cells (Figure 2A). In addition, we examined the physical association between Pak1 and CaMKII by co-immunoprecipitation assays. HEK293T cells co-transfected pCMV6M-Pak1 and pEGFP-CaMKII expression vectors were lysed and CaMKII was immunoprecipitated with anti-GFP antibodies. The immunoprecipitates were separated by acrylamide gel electrophoresis and probed for associated Pak1 by Western blot analysis. The presence of Pak1 was readily detectable upon probing the immunoblots with the anti-myc antibody in immunoprecipitates (Figure 2B). Reciprocal immunoprecipitation of myc-Pak1 with the anti-myc antibody, was probed for associated CaMKII with the anti-GFP antibody (Figure 2B). Next, we confirmed the interaction of endogenous Pak and CaMKII in breast cancer cells. Since the western blot analysis shown in Supplementary Figures S1C showed that MCF7, SK-BR3 and MDA-MB-231 cells co-express high amounts of both proteins, we analyzed their co-localization and ability to interact by immunofluorescence and co-immunoprecipitation (Figures 2C,D). Altogether, our results indicate that CaMKII is a novel substrate of Pak1, and that both proteins physically interact in a cellular context.

### Pak1 and CaMKII Are Coordinately Overexpressed in Human Breast Cancer Samples

**FIGURE 3:**
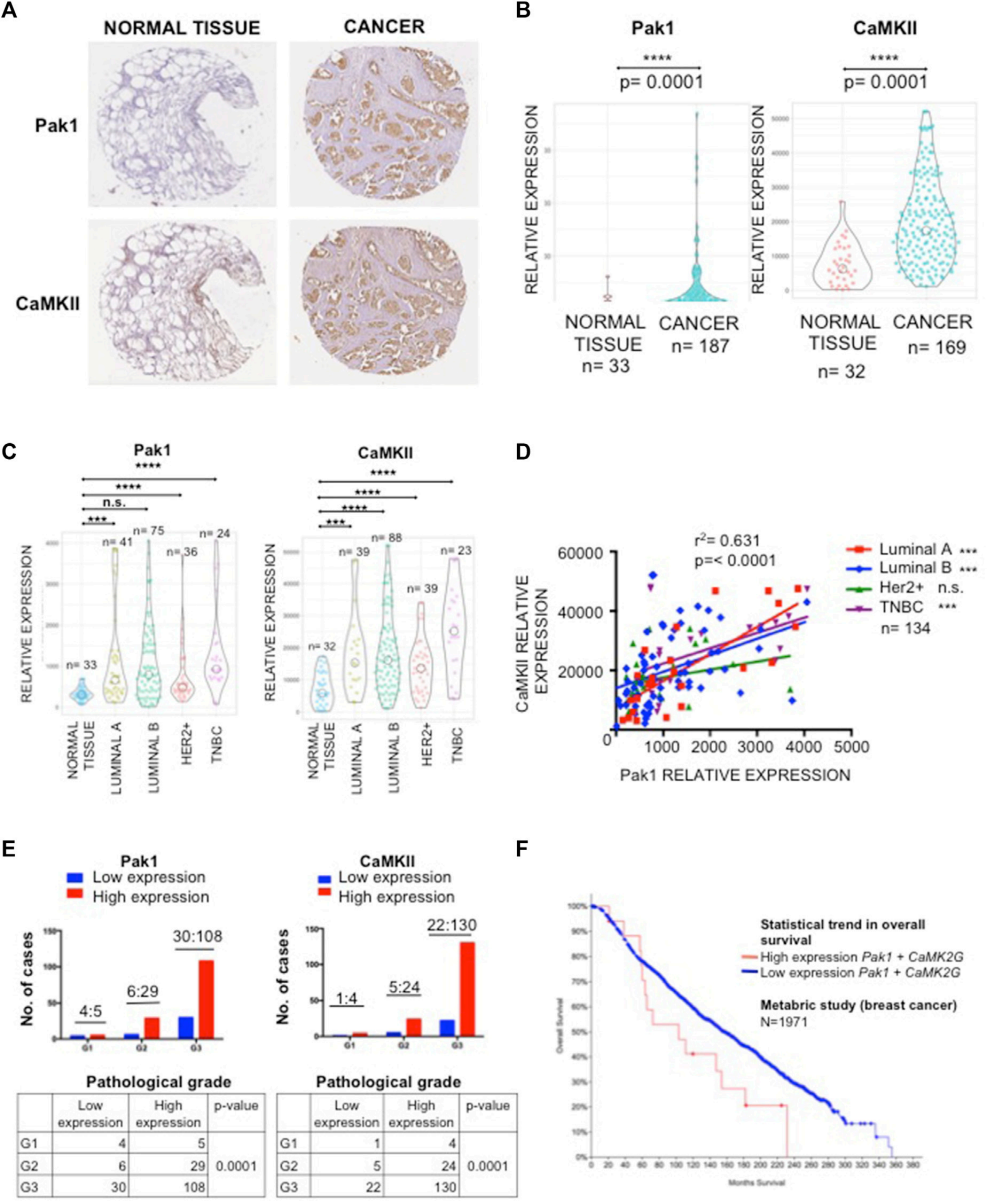
Correlation of immunohistochemical staining of Pak1 and CaMKII in human breast cancer. **(A)** Representative example of human breast cancer specimens from TMA that stained positive or negative for Pak1 (upper panel). Matching specimens from the same patient are shown for CaMKII staining (bottom panel). **(B)** Violin charts comparing the relative expression of Pak1 and CaMKII between breast tumor samples and adjacent normal tissues. **(C)** Violin charts comparing the relative expression of Pak1 and CaMKII between the different intrinsic breast cancer subtypes (Luminal A, Luminal B, Her2 positive, and Triple Negative) tumor samples and adjacent normal tissues. **(D)** Correlation between Pak1 and CaMKII expression in the different intrinsic breast cancer subtypes, the *X* and *Y* axis represent the integrated optical density (region score) of immunohistochemical staining intensity. **(E)** Association of clinicopathological features with Pak1 (left panel) and CaMKII expression (right panel). The expression of Pak1 and CaMKII was significantly associated with histological grade (****p < 0.0001). **(F)** Kaplan-Meier curves according to Pak1 and CaMKII co-expression status for overall survival. Data form a metabric study shows that patients with high expression levels of Pak1 and CaMKII have worst overall survival than patients with low expression levels of these proteins.

To investigate the protein expression patterns of Pak1 and CaMKII in human breast cancer, we used a TMA containing normal and tumor samples for IHC staining. Overall, both Pak1 and CaMKII showed higher expression levels in tumor samples than in non-transformed adjacent tissue (*p* < 0.0001) (Figures 3A,B). To more thoroughly examine the relationship between Pak1 and CaMKII expression in the four different tumor intrinsic subtypes, the tumor samples were stratified according to ER, PR and Her2 status. Here, we found that the relative expression level of CaMKII in the four breast cancer intrinsic subtypes is higher than in normal breast tissues. Regarding Pak1, we found that the expression level of this kinase is elevated in Luminal A, Luminal B and TNBC tumor samples. However, it is not statistically significant in Her2 positive tumors when compared to normal tissue (*p* < 0.0001) (Figure 3C). Next, we evaluated the relationship between the expression levels of Pak1 and CaMKII using a Spearman rank correlation. Overall, we found a strong correlation between Pak1 and CaMKII expression in Luminal A, Luminal B and TNBC tumor samples (*r* ^2^ = 0.631, *p* < 0.0001) (Figure 3D). Furthermore, we observed that the expression of Pak1 and CaMKII was significantly associated with histological grade (*p* < 0.0001) (Figure 3E). Finally, we assessed whether Pak1 and CaMKII*γ* expression is associated with breast cancer patient outcome by examining the expression level of both genes at mRNA level from a set of data of a breast cancer METABRIC study obtained from the Cancer Genome Atlas website. The result of this analysis indicates that high Pak1 and CAMKII*γ* expression is associated with significantly worse overall survival. In contrast, patients with low expression levels of Pak1 and CAMKII*γ* have a better prognosis (Figure 3F). Altogether, our results indicate that Pak1 and CaMKII are co-expressed in human Luminal and TNBC specimens, and that high expression of these proteins is associated with significantly worse overall survival.

### *In vitro* Synergy Between Pak and CaMKII Inhibitors

**FIGURE 4:**
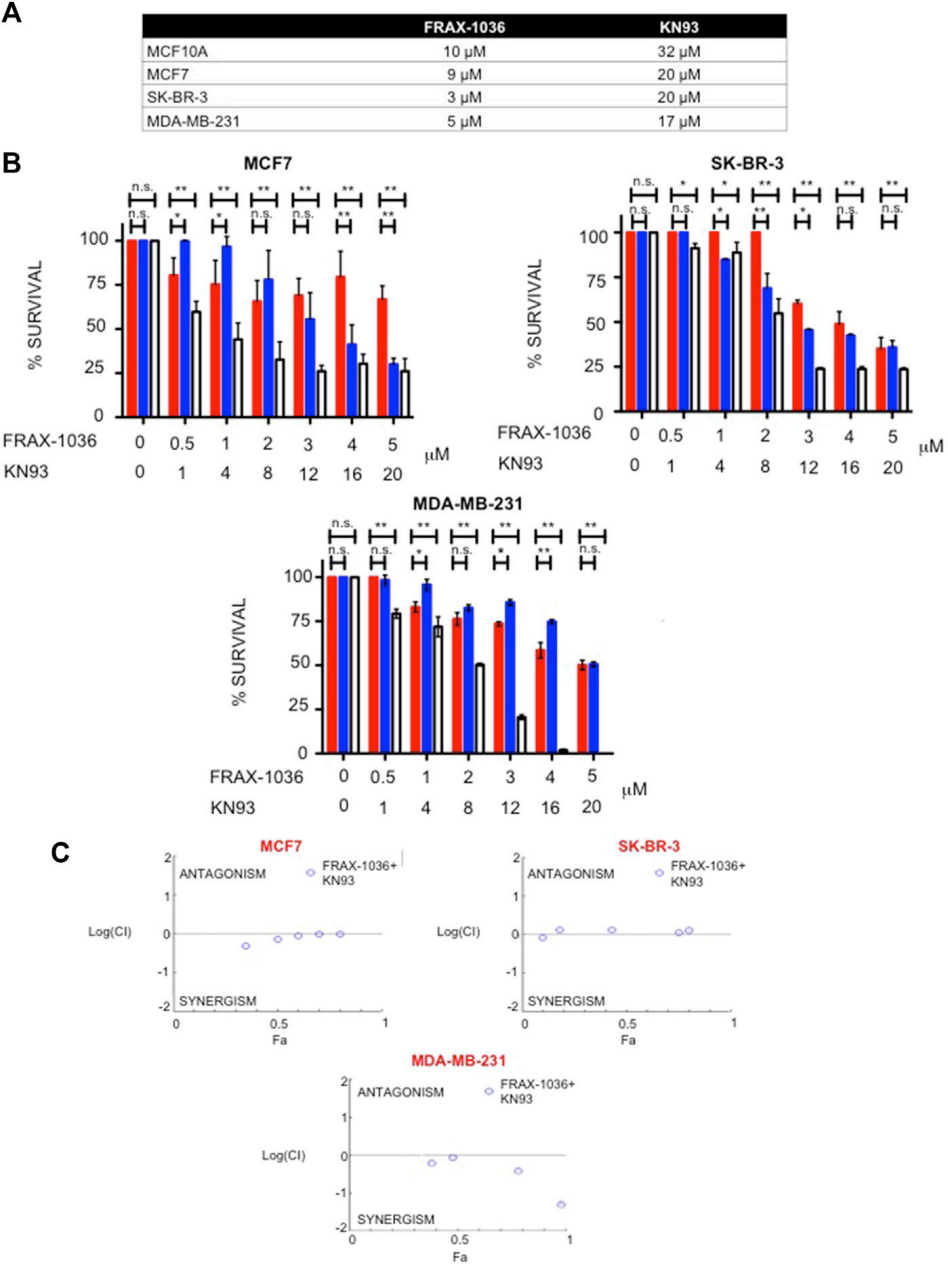
Synergistic interactions between Pak and CaMKII inhibitors. **(A)** Effect of Pak and CaMKII inhibitors on survival of MCF10A, MCF7, SK-BR3 and MDA-MB-231cells. Cells were treated with varying amounts of FRAX-1036 or KN93 for 72 h; cell viability was determined by Trypan blue exclusion and the IC_50_ was determined. **(B)** Effect of Pak and CaMKII combined inhibition on survival of breast cancer cells. MCF7, SK-BR-3, and MDA-MB-231 cells were treated with the indicated amounts of FRAX-1036 (red bars), KN93 (blue bars) or both inhibitors (with bars) for 72 h; cell viability was determined by Trypan blue exclusion. **(C)** CI curve analysis for FRAX-1036 plus KN93 in MCF7, SK-BR-3 and MDA-MB-231 indicates synergy. CI values less than, equal to, or greater than 1 indicate synergy, additive effect or antagonism respectively.

As Pak1 and CaMKII are co-expressed and interact in human breast cancer cells, we tested the effect of small-molecule inhibitors of these kinases, alone and in combination, on the survival of Luminal, Her2 positive and TNBC cells. These compounds included FRAX-1036, which is a potent and selective inhibitor of Group I Paks (Ong et al., 2015), and KN93, a selective CaMKII inhibitor that impedes the binding of the CaM/Ca^2+^ complex to CaMKII preventing its activation (Sumi et al., 1991). The non-transformed MCF10A cells and the breast cancer cell lines MCF7, SK-BR3 and MDA-MB-231 were treated with varying concentrations of FRAX-1036 or KN93 and the effect on cell survival was assessed after 72 h of treatment. The results showed that SK-BR3 and MDA-MB-231 cells were more sensitive to both small-molecule inhibitors than the non-transformed MCF10A cells and the luminal MCF7 breast cancer cells. However, breast cancer cells were more sensitive to FRAX-1036 than to KN93. The IC_50_ values of FRAX-1036 in MCF10A, MCF7, SK-BR3 and MDA-MB-231 cells were 10, 9, 3, and 5 μM respectively; the IC_50_ values for KN93 were 32, 20, 20, and 17 μM respectively (Figure 4A). Since Pak1 and CaMKII expression is elevated in Luminal, Her2 positive and TNBC cells, we performed drug synergy test, in MCF7, SK-BR-3, MDA-MB-231 cells. Our results showed that Pak and CaMKII inhibition was synergistic in all the breast cancer cell lines (Figures 4B,C).

Next, we validated by western blot that the patient derived TNBC cell line MBCDF-B4 (Esparza-López et al., 2016) expressed Pak1 and CaMKII, and that both proteins interacted by co-immunoprecipitation (Supplementary Figures S2A,B). Then, varying concentrations of FRAX-1036 and KN93 were coadministred to MBCDF-B4 cells, and a potent synergistic effect was noted (Supplementary Figures S2C,D). Finally, in order to evaluate if the coadministration of both small-molecule inhibitors induce cell death and/or cell cycle arrest, we performed a Caspase-Glo 3/7 assay. Our results showed that the combined inhibition of Pak and CaMKII did not merely produce cytostasis, but also resulted in cell death, increasing the frequency of apoptosis by nearly a factor of 5 in SK-BR-3 and MDA-MB-231 cells and a factor of 15 in MBCDF-B4 cells. In contrast, MCF7 cells displayed a modest but statistically significant effect (Supplementary Figure S2E).

### Pak and CaMKII Combined Inhibition Suppresses Proliferation and Induces Apoptosis in 3D Cell Cultures of Breast Cancer Cells

**FIGURE 5:**
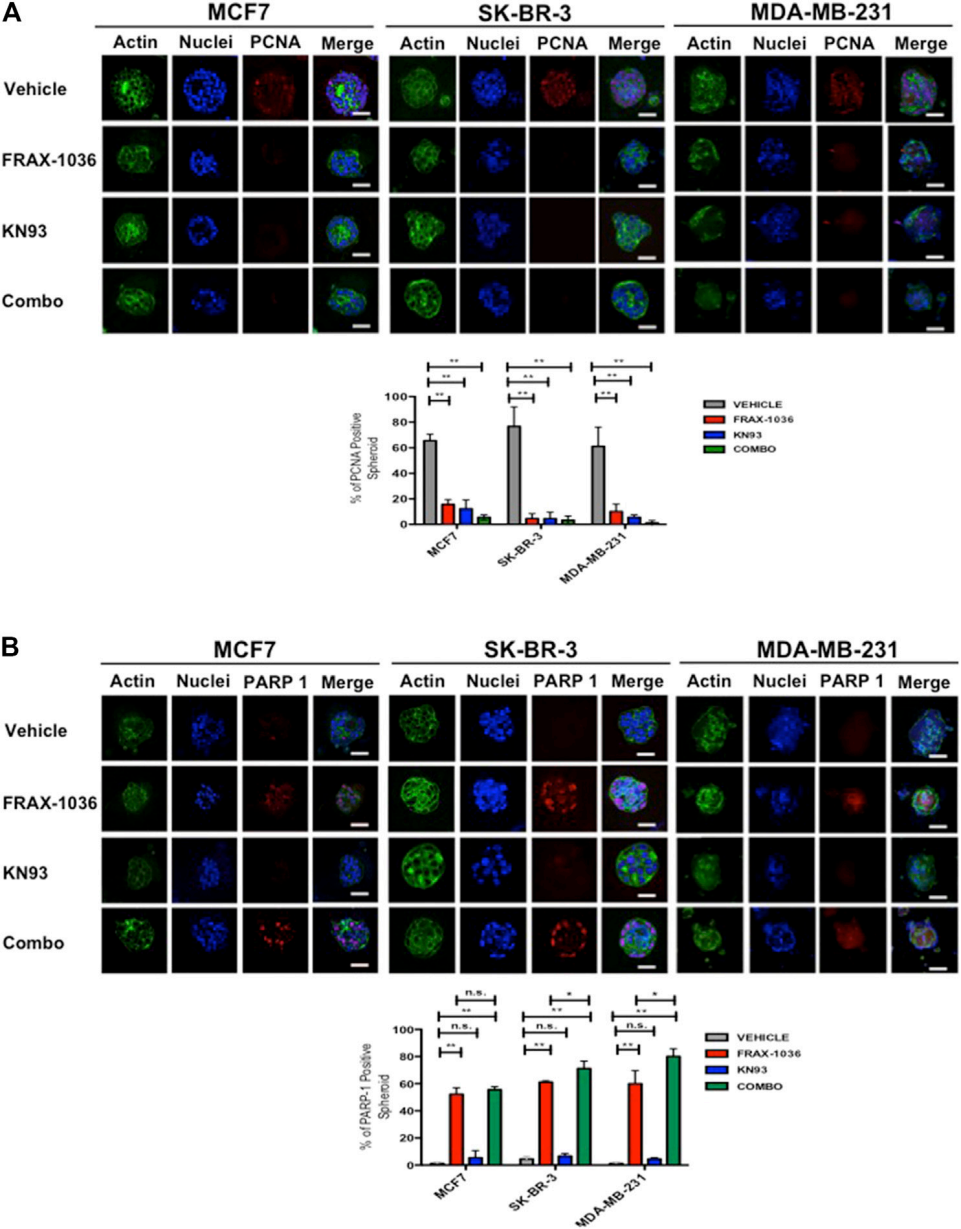
Pak and CaMKII pharmacological inhibition reduces cell proliferation and induces apoptosis in breast cancer cells 3D cultures. MCF7, SK-BR-3 and MDA-MB-231 cells were grown for 7 days in matrigel to form spheroids and incubated in medium (vehicle) or in medium containing 1 µM of FRAX-1036, 4 µM of KN93 or 0.5:1 µM of FRAX-1036 and KN93 respectively for 5 days. The structure of spheroids was visualized by incubation with DAPI (blue) and Oregon Green Phalloidin (green). To determine the effect of Pak and CaMKII inhibition on cell proliferation **(A)** and apoptosis **(B)**, the 3D cultures were incubated with anti-PCNA **(A)** or anti cleaved PARP-1 antibodies. The number of PCNA or PARP-1 positive nuclei in spheroids were taken as indicators of cell proliferation or apoptosis, respectively. Results are shown as mean ± S.E. and the differences between MCF-7, SK-BR-3, and MDA-MB-231 spheroids were shown to be statistically significant (**p < 0.01). Scale bar, 50 μm. n. s., not statistically significant.

In order to evaluate the effect of Pak and CaMKII pharmacological inhibition in tumor growth, we used a 3D cell culture system that closely resemble an *in vivo* cell environment and mimics cell-cell and cell-matrix interactions that exist in living tissues. To this end, MCF7, SK-BR-3 and MDA-MB-231 cells were grown for 7 days atop a reconstituted layer of Matrigel to form spheroids and incubated in medium supplemented with vehicle, the Pak or CaMKII inhibitors alone, or medium supplemented with both drugs for 5 days. Next, we stained the spheroids with antibodies directed against PCNA or cleaved PARP-1 in order to evaluate the effect of Pak and CaMKII inhibition on proliferation or apoptosis respectively (Figure 5). Our results showed that the MCF7, SK-BR-3 and MDA-MB-231 spheroids treated with vehicle were positive for PCNA (63, 79, and 60% respectively). In contrast, the FRAX-1036 and KN93 treated spheroids displayed a dramatic reduction in the expression of PCNA. Finally, the coadministration of both inhibitors was more effective in SK-BR-3 and MDA-MB-231 cells, where only the 6 and 1% of the spheroids analyzed were positive for PCNA expression (Figure 5A). These results indicate that combined inhibition of Pak and CaMKII reduces cell proliferation in breast cancer 3D cultures.

To determine if in the 3D cell culture setting the small-molecule inhibitors targeting Pak or CaMKII has a cytotoxic or a cytostatic effect, we also quantified the percent of positive cleaved-PARP-1 spheroids. Here, we observed that the MCF7, SK-BR-3 and MDA-MB-231 spheroids treated with vehicle were negative for cleaved-PARP-1 staining, and according to the aforementioned results, CaMKII inhibition has a cytostatic effect. In contrast Pak inhibition induced apoptosis in MCF7, SK-BR-3 and MDA-MB-231 cells, where the 53, 60, and 56% of the spheroids respectively, were positive for cleaved-PARP-1 staining. Notably, the coadministration of Pak and CaMKII inhibitors induced a significant increase in apoptosis only in SK-BR-3 and MDA-MB-231 cells, where the 70 and 76% of the spheroids analyzed were positive for cleaved-PARP-1 staining (Figure 5B). Overall, our results suggest that Pak and CaMKII combined inhibition has a more potent effect in Her2 positive and TNBC cells. Therefore, we used MDA-MB-231 cells and the patient derived TNBC cell line MBCDF-B4 (Esparza-López et al., 2016) for further experiments.

### Pak1 and CaMKII Combined Inhibition Suppresses Migration and Invasion in TNBC Cells

**FIGURE 6:**
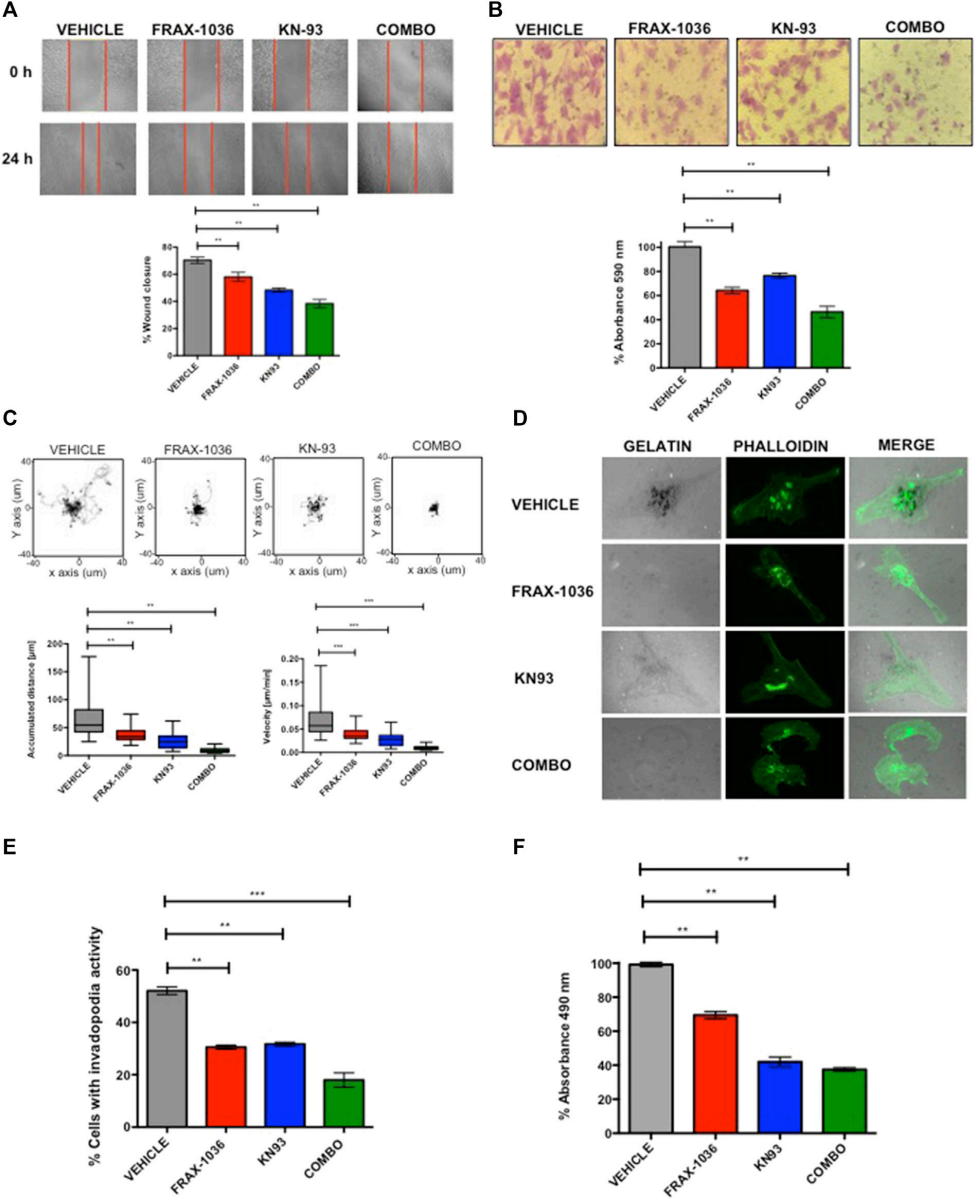
Combined Pak and CaMKII pharmacological inhibition impairs cell migration and invasion in breast cancer cells. **(A)** Representative images from in vitro scratch assay, MDA-MB-231 cells were treated with vehicle, 1 µM of FRAX-1036, 4 µM of KN93 or 0.5:1 µM of FRAX-1036 and KN93 respectively (upper panel). The quantitative evaluation and statistical analysis of wound closure percentage was calculated with ImageJ software (bottom panel). Results are expressed as means ± SD of three experiments (*p < 0.05). **(B)** Representative images from cell directional migration assay, MDA-MB-231 cells were treated with vehicle, or the aforementioned amounts of FRAX-1036, KN93 or the combination of both small-molecule inhibitors (upper panel). The bar graphic shows quantitative analysis of crystal violet extracted from migratory cells (bottom panel). Results are expressed as means ± SD of three experiments (*p < 0.05). **(C)** Random migration of MDA-MB-231 cells treated with FRAX-1036 and/or KN93 was monitored by microscopy. Representative track plots from at least three independent experiments are shown. Bar graphs show comparisons of accumulated distance and migration speed. Data are presented as means ± SEM. **(D)** MDA-MB-231 cells were treated as before and plated on Alexa594-gelatin coverslips for 4 h, fixed permeabilized, and stained for F-actin. **(E)** The percentage of cells forming invadopodia, based on degradation of the fluorescent gelatin, was quantified using confocal microscopy. Data are presented as means ± SEM. **(F)** MDA-MB-231 cells were treated with 1 µM of FRAX-1036, 4 µM of KN93 or 0.5:1 µM of FRAX-1036 and KN93 respectively, stimulated with 5 μM of LPA and RhoA activity was analyzed by G-LISA assays. Data are presented as means ± SEM.

As Pak and CaMKII activation have been strongly associated with enhanced cell motility and invasion (Arias-Romero and Chernoff, 2008; Radu et al., 2014; Chi et al., 2016), we next tested the effects of these small molecule inhibitors on these processes. First, using a wound-healing model and a transwell, we found that a sub-lethal dose of FRAX-1036 or KN93 reduced the motility of MDA-MB231 cells (Figures 6A,B) and MBCDF-B4 cells (Supplementary Figures S3A,B). Moreover, the coadministration of both inhibitors drastically reduced the migration of both cell lines. Notably, the MBCDF-B4 were much more sensitive to the combined inhibition of Pak and CaMKII than MDA-MB-231 cells (Supplementary Figures S3A,B). Next, we performed cell-tracking analyses in order to determine the directional movement and speed of cells treated with Pak and CaMKII inhibitors alone or in combination. We observed that cells treated with FRAX-1036 or KN93 migrate slower than control cells. However, cells co-treated with both inhibitors remained viable, but did not migrate. In addition, the ratio of accumulated distance and velocity was dramatically reduced in the cells co-treated with anti-Pak and anti-CaMKII agents (Figure 6C; Supplementary Figure S3C).

In order to define the role of Pak and CaMKII in the invasive potential of TNBC cells, we used the invadopodia assay, where the areas of gelatin degradation were co-localized with actin puncta as markers of invadopodia formation (Md Hashim et al., 2013). We found that pharmacological inhibition of either Pak or CaMKII in MDA-MB-231 cells resulted in a 50% reduction in invadopodia formation compared to control cells, where as the combined inhibition of these kinases resulted in a more pronounced effect (Figures 5D,E). The role of Pak kinases and Rho GTPases in invadopodia formation in breast cancer cells has been extensively documented (Nakahara et al., 2003; Ayala et al., 2008). However the role of CaMKII in this cellular event is unknown. Since CaMKII promotes neurite extension through the activation of the small GTPase RhoA in hippocampal neurons (Fink et al., 2003), we hypothesized that CaMKII might promote cytoskeletal rearrangements in TNBC cells in a RhoA–dependent manner. To determine whether CaMKII inhibition impairs RhoA activity in TNBC cells, we performed a RhoA G-LISA activation assay (Figure 6F). Our results indicate that MDA-MB-231 cells treated with FRAX-1036 had a 30% reduction of RhoA activity compared to control cells. In contrast, MDA-MB-231 cells treated with KN93 had more than 60% reduction in RhoA activity. Surprisingly, the combined inhibition of Pak and CaMKII had similar effects in terms of RhoA activity reduction than the inhibition of CaMKII alone. Altogether, our results suggest that a Pak/CaMKII/RhoA axis is important for migration and invasion of TNBC cells.

### Inhibition of Tumor Growth by Small-Molecule Inhibitors of Pak and CaMKII

**FIGURE 7:**
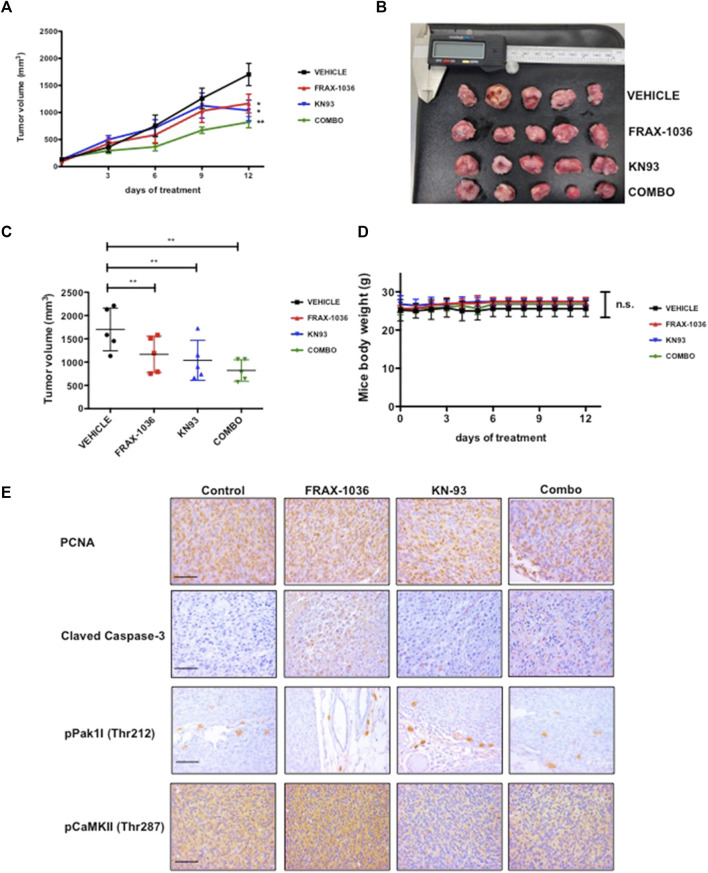
Inhibition of Pak and CaMKII impedes the tumorigenicity of triple negative breast cancer cells. MDA-MB-231 cells were injected into the flanks of BALB/c-nu/nu mice. Ten days after innoculation, the animals were treated with vehicle or inhibitors for 12 days. **(A)** and **(B)** Volumetric changes in tumor size between untreated mice (vehicle) and mice treated with inhibitors, data presented as mean ± SEM. **(C)** Average tumor size of untreated mice (vehicle) and mice treated with inhibitors, data presented as mean ± SEM. **(D)** Changes in the weight of the mice on treatment relative to the initial weight, data presented as mean ± SEM. **(E)** Representative example of tumor sections between untreated mice and mice treated with Pak inhibitor; CaMKII inhibitor and a combination of Pak and CaMKII inhibitors stained for PCNA, Cleaved Caspase-3, phospho-Pak1 and phospho-CaMKII.

We next proved the effects of these small-molecule inhibitors on the growth of MDA-MB-231 in a xenograft setting. MDA-MB-231 cells were xenografted to BALB/c-nu/nu mice and tumors were allowed to form for 10 days. The mice were then treated with vehicle, Pak inhibitor FRAX-1036, CaMKII inhibitor KN93, or FRAX-1036 plus KN93, for 12 days. Tumor volumes were measured with a digital caliper every 3 days, at which time the animals were culled. Treatment with either FRAX-1036 or KN93 had a marked negative effect on tumor growth, yielding tumors of about one-half the volume of tumors in untreated animals. Interestingly, animals treated with the combined Pak and CaMKII inhibitors showed a dramatic effect on tumor growth yielding tumors of less than one-third the volume of tumors in untreated mice (Figures 7A–C). All therapies were well tolerated, with weight of drug- and vehicle-treated mice not significantly differing (Figure 7D).

We then analyzed the effect of Pak and CaMKII targeted therapy in xenograft tumors by IHC. Our results revealed that FRAX-1036 treatment prevented cell-cycle progression and induced apoptosis (Figure 7E). Consistent with our previous observations (Figure 5; Supplementary Figure S2C) and previous reports (Yuan et al., 2007), KN93, had a cytostatic effect, without any detectable changes in Caspase-3 activity when compared to the vehicle group. In contrast, when coadministered, the inhibitors blocked cell-cycle progression and caused extensive apoptosis (Figure 5E). Together, our results provide evidence that dual inhibition of Pak1 and CaMKII may be useful for the treatment of TNBC.

## Discussion

Recent evidence suggests that Pak1 plays an important role in the development and progression of human breast cancer (Kanumuri et al., 2020; Kumar et al., 2020). For example, *pak1* gene is commonly amplified, and its elevated expression has been is associated with tamoxifen-resistant disease (Holm et al., 2006). In addition, recent reports indicate that Pak1 overexpression is associated with poor prognosis in a number of different solid tumors, with higher Pak1 expression linked to poor outcome including shortened progression free and overall survival (Fang et al., 2016). However, the molecular mechanisms that explain the contribution of Pak1 and its downstream targets to breast carcinogenesis are not completely understood.

In this study, we identified CaMKII as a new Pak1 substrate in breast cancer cells. This conclusion is supported by the ability of Pak1 to phosphorylate *in vitro* the residues T277 and T287 located in the regulatory domain of CaMKII, and to co-localize and interact with it in co-immunoprecipitation assays. Furthermore, our results indicate that Pak1 and CaMKII expression is correlated in human breast cancer specimens, and our analysis of the METABRIC study showed significantly worse overall survival in breast cancer patients with co-expression of Pak1 and CaMKII. Correspondingly, we observed that combined inhibition of Pak1 and CaMKII has a potent synergistic effect in Her2 positive and TNBC cells *in vitro,* and may represent an attractive therapeutic target for the treatment of breast cancer*.* The synergistic effect observed in Her2 positive cells was not surprising, due to the fact that Pak1 is an essential mediator of Her2 signaling in mammary tumors, and some reports suggest that combined inhibition of Pak1 and some other relevant oncogenes such as BCR-ABL1, Akt, *β*-catenin and Aurora A might be useful for treating cancers driven by oncogenes for which Pak1 is thought to be an obligate signaling element (Arias-Romero et al., 2013; Walsh et al., 2013; Flis et al., 2019; Korobeynikov et al., 2019). However, since TNBC represents a challenge for clinicians due to its poorer prognosis, lack of targeted therapies and high mortality in comparison to other breast cancer subtypes, we decided to examine the effects of Pak and CaMKII combined inhibition in the survival of TNBC cells. To date, there are few studies about the role of PAK1 in TNBC. Recently, Shi et al. (2021) showed that sphingosine kinase 2 (SphK2) promotes metastasis of TNBC cells through a Pak1/LIMK1/Cofilin signaling pathway (Shi et al., 2021). In addition, targeting Pak1 with liposomes containing the allosteric Group I Paks inhibitor, IPA-3, reduced cell viability and induced apoptosis in metastasic in TNBC cells (Najahi-Missaoui et al., 2020). Given the roles of Pak1 and CaMKII in regulating cytoskeletal rearrangements (Fink et al., 2003; Arias-Romero et al., 2010), and that small-molecule inhibitors targeting both Pak and CaMKII impairs cell migration in several types of cancer (Dai L. et al., 2015; Chen et al., 2017; Semenova et al., 2017; Araiza-Olivera et al., 2018; Chow et al., 2018; Korobeynikov et al., 2019; Najahi-Missaoui et al., 2020; Sim et al., 2020; Shi et al., 2021), we speculated that the combined inhibition of these two kinases may have more adverse effects in cell motility and invasion than the monotherapy against any of these enzyme in TNBC cells. Our results indicate that co-administration of Pak and CaMKII inhibitors impairs not only the intrinsic migratory behavior of TNBC cells, but their capability to form invadopodia and subsequently degrade gelatin matrix. The invadopodia assay has been extensively used as an indicator of the invasive potential of cancer cells. The role of Pak1 during invadopodia formation and maturation has been described (Gasparski et al., 2019), and although the involvement of CaMKII in this cellular event is unknown, it is well documented that CaMKII activity promotes actin polymerization and neurite outgrowth in a RhoA dependent-manner (Fink et al., 2003), suggesting that in our model, invadopodia formation may be regulated in part by a Pak1/CamKII/RhoA signaling pathway. Finally, the results of our xenograft setting showed a significant delay in tumor growth when the mice were treated with small-molecule inhibitors of Pak and CaMKII. Overall, our results suggest that combined inhibition of Pak and CaMKII may provide a new therapeutic strategy for the treatment of TNBC.

## Data Availability

Publicly available datasets were analyzed in this study. This data can be found here: EGAC00001000484. The data of the phospho-antibody microarray presented in this study are deposited in the GEO repository, accession number GSE192492.
