# Supplementary material for: p21-Activated Kinase 1 Promotes Breast Tumorigenesis via Phosphorylation and Activation of the Calcium/Calmodulin-Dependent Protein Kinase II
Source: Front Cell Dev Biol. 2022 Jan 17;9:759259. doi: 10.3389/fcell.2021.759259 (PMC8802317; doi:10.3389/fcell.2021.759259)
Supplement: Supplementary file 1 [file DataSheet1.docx]

**
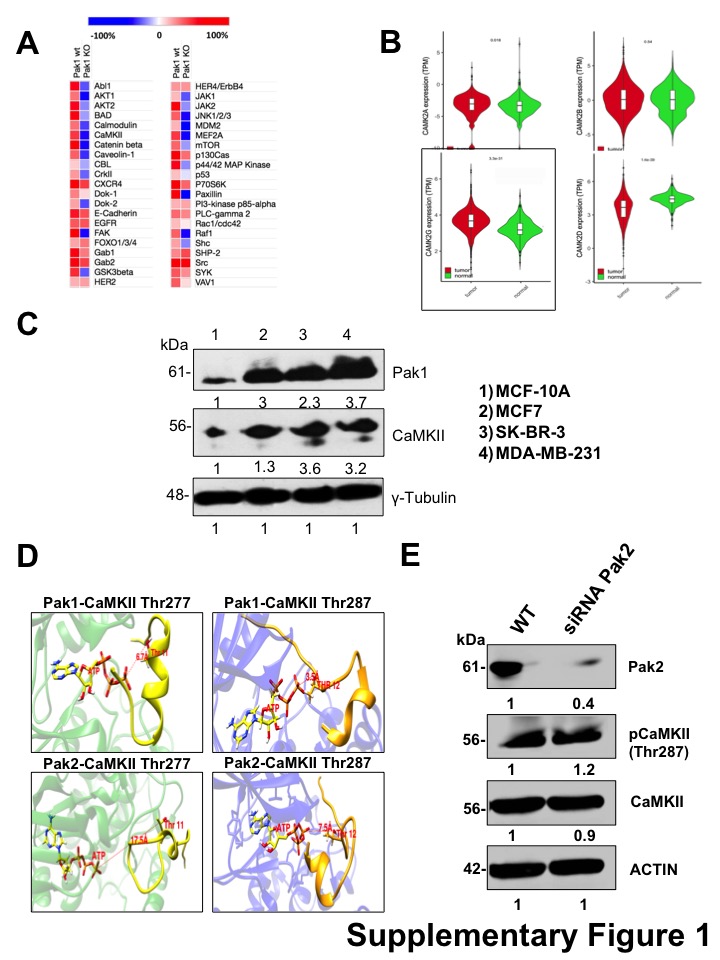
**

**Supplementary Figure 1. CaMKII is co-expressed and phosphorylated by Pak1 in breast cancer cells.** (A) Heat map of phospho-protein array, the activities of a panel of signaling proteins were assessed using a phospho-antibody array. Results are presented as changes in phosphorylation between control and Pak1-deficient cells. (B) Violin charts comparing the relative expression at mRNA level the four members of the CaMKII family in human breast cancer specimens. (C) Pak1 and CamKII protein expression in human breast cancer cell lines was assessed by western blot. γ-Tubulin was used as loading control. (D) Visualization of the complex of peptide I (left), and peptide II (right) with ATP-bound Pak1 and Pak2. The boxed panels show a closer view of the O_(Thr)_-γP interaction for peptides I and II, respectively. (E) Pak2 knock down with siRNAs does not affect the activation of CaMKII in breast cancer cells. Numbers indicate fold expression or phosphorylation change in CaMKII relative to control cells.

**
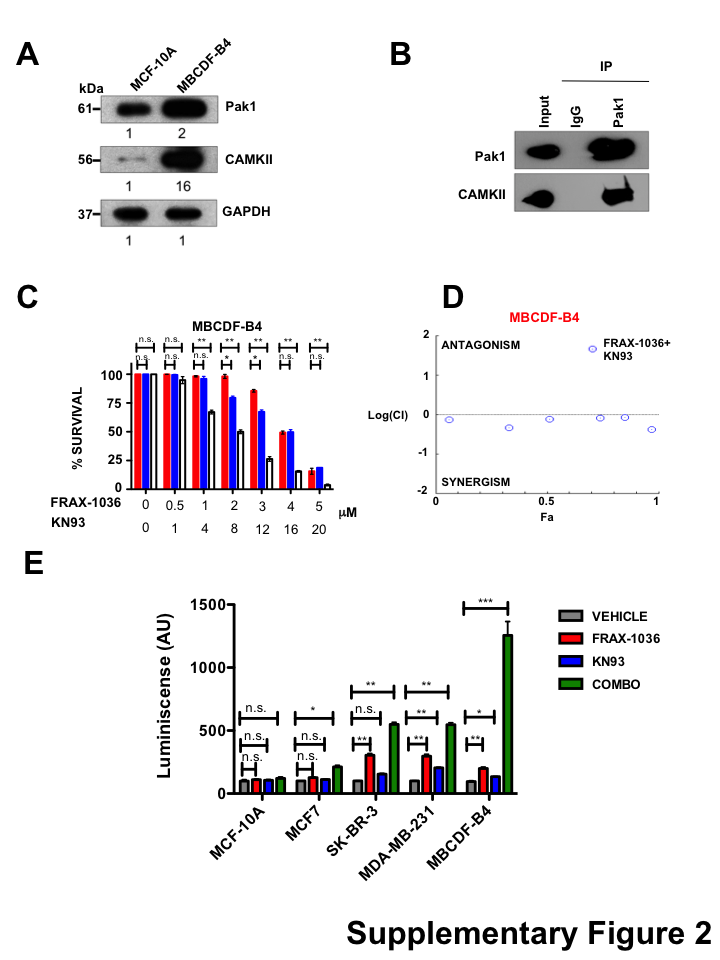
**

**Supplementary Figure 2. .** (A) Pak1 and CaMKII expression in the non-transformed MCF10A cell line and the patient derived TNBC cell line MBCDF-B4 was assessed by western blot. GAPDH was used as loading control. Numbers indicate fold expression change relative to control cells. (B) Coimmunoprecipitation of endogenous Pak1 and CaMKII. MBCDF-B4 cell lysates were subjected to immunoprecipitation with anti-Pak1, anti-CaMKII or isotype control IgG antibodies. The presence of Pak1 and CaMKII in cell extracts prior to immunoprecipitation was assessed using specific antibodies (Input). (C) Effect of Pak and CaMKII combined inhibition on survival of breast cancer cells. MBCDF-B4 cells were treated with the indicated amounts of FRAX-1036 (red bars), KN93 (blue bars) or both inhibitors (white bars) for 72 hours; cell viability was determined by Trypan blue exclusion. (C) CI curve analysis for FRAX-1036 plus KN93 in MBCDF-B4 indicates synergy. CI values less than, equal to, or greater than 1 indicate synergy, additive effect or antagonism respectively. (E) Effect of Pak and CaMKII inhibitors on apoptosis induction of MCF10A, MDA-MB-231 and MBCDF-B4 cells. Cells were treated with FRAX-1036 and/or KN93 for 16 hours. The activity of caspase-3/7 was measured by the Caspase-Glo 3/7 assay.


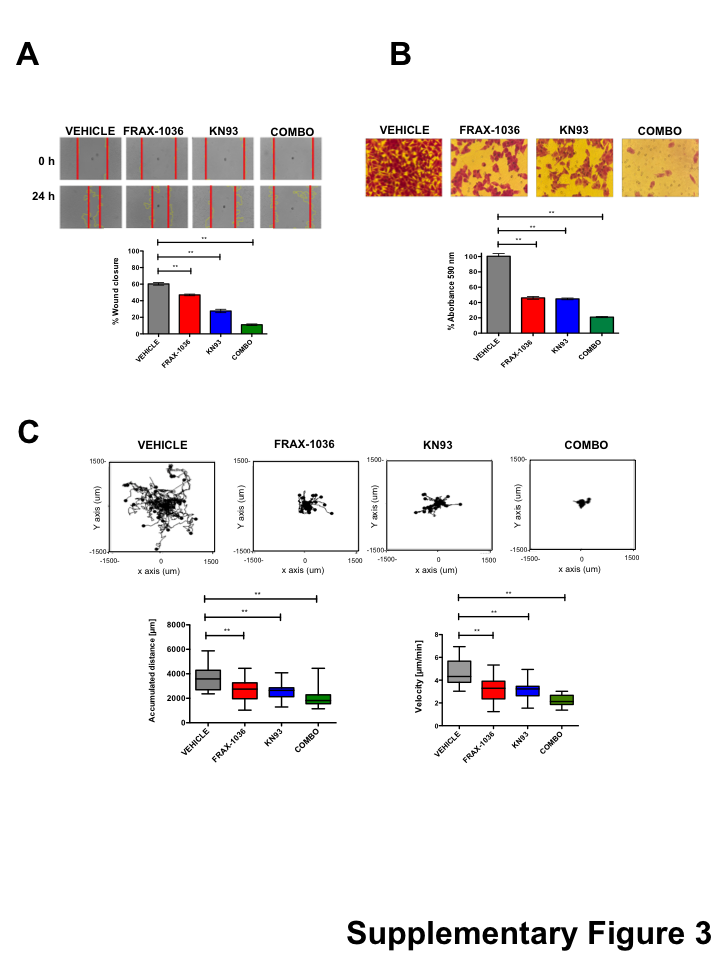


**Supplementary Figure 3. Pak1 and CaMKII interact in a patient derived TNBC cell line, and their combined inhibition is synergistic.** (A) Representative images from in vitro scratch assay, MBCDF-B4 cells were treated with vehicle, FRAX-1036, KN-93 or the combination of both small-molecule inhibitors (upper panel). The quantitative evaluation and statistical analysis of wound closure percentage was calculated with Image J software (bottom panel). Results are expressed as means ± SEM of three experiments (*p<0.05). (B) Representative images from cell directional migration assay, MBCDF-B4 cells were treated with vehicle, FRAX-1036, KN93 or the combination of both small-molecule inhibitors (upper panel). The bar graphic shows quantitative analysis of crystal violet extracted from migratory cells (bottom panel). Results are expressed as means ± SEM of three experiments (*p<0.05). (C) Random migration of MBCDF-B4 cells treated with FRAX-1036 and/or KN93 was monitored by microscopy. Representative track plots from at least three independent experiments are shown. Bar graphs show comparisons of accumulated distance and migration speed. Data are presented as means ± SEM.
